# DGDTA: dynamic graph attention network for predicting drug–target binding affinity

**DOI:** 10.1186/s12859-023-05497-5

**Published:** 2023-09-30

**Authors:** Haixia Zhai, Hongli Hou, Junwei Luo, Xiaoyan Liu, Zhengjiang Wu, Junfeng Wang

**Affiliations:** School of Software, Henan Polytechnic University, Jiaozuo, 454003 China

**Keywords:** Drug–target binding affinity, Dynamic graph attention network, Long short-term memory, Drug discovery

## Abstract

**Background:**

Obtaining accurate drug–target binding affinity (DTA) information is significant for drug discovery and drug repositioning. Although some methods have been proposed for predicting DTA, the features of proteins and drugs still need to be further analyzed. Recently, deep learning has been successfully used in many fields. Hence, designing a more effective deep learning method for predicting DTA remains attractive.

**Results:**

Dynamic graph DTA (DGDTA), which uses a dynamic graph attention network combined with a bidirectional long short-term memory (Bi-LSTM) network to predict DTA is proposed in this paper. DGDTA adopts drug compound as input according to its corresponding simplified molecular input line entry system (SMILES) and protein amino acid sequence. First, each drug is considered a graph of interactions between atoms and edges, and dynamic attention scores are used to consider which atoms and edges in the drug are most important for predicting DTA. Then, Bi-LSTM is used to better extract the contextual information features of protein amino acid sequences. Finally, after combining the obtained drug and protein feature vectors, the DTA is predicted by a fully connected layer. The source code is available from GitHub at https://github.com/luojunwei/DGDTA.

**Conclusions:**

The experimental results show that DGDTA can predict DTA more accurately than some other methods.

## Background

Drug–target interaction (DTI) prediction is a critical task in drug discovery and drug repositioning [1, 2]. Structural changes to a drug can significantly alter its binding affinity with proteins [3], making it important to predict whether a drug can bind to a specific protein. However, the traditional high-throughput screening experiments used to detect this activity are expensive and time-consuming [4]. Therefore, computing methods for DTI prediction have become popular and effective [5, 6].

DTI calculation methods focus on binary classification [2, 7], and the main goal is to determine whether a drug and a target interact with each other [8]. However, the assumed binding strength values of the given protein and the drug compound are continuous and referred to as their binding affinity. The drug–target pair prediction task is described as an affinity prediction problem [8] in which, the binding affinity score is directly used, thus creating a more realistic experiment. In addition, regression-based models are more advantageous in approximating the strength of DTIs [9], making them more conducive to the discovery of new drug compounds in the limited drug research space.

Recently, some methods [10, 11] for predicting drug–target affinity (DTA) have been developed. SimBoost [11] enhances the performance of learning-based methods by extracting features from drugs, targets, and drug–target pairs and providing them to gradient-enhanced supervised learning methods. Affinity is characterized by an inhibition constant (${K}_{i}$), dissociation constant (${K}_{d}$), changes in free energy measures ($\delta G$,$\delta H$), half-maximal inhibition constant ($I{C}_{50}$) [12], half-maximal activity concentration ($A{C}_{50}$) [13], KIBA score [14] and scoring. Stronger affinity readings indicate greater DTIs [15]. In the KronRLS [10, 16] model, the Kronecker products of a drug and target are constructed by drug and protein pairs to calculate the kernel K of the pairs, which is entered into a regularized least-squares regression model (RLS) to predict the binding affinity.

With the success of deep learning, various deep networks have been used for DTA prediction [8, 13], and have achieved better performance than machine learning. Some prediction methods are summarized in Table 1. In the DeepDTA [8] model, one-dimensional sequences of drugs and proteins are fed into a convolutional neural network (CNN) to extract the features of drugs and their targets through the (simplified molecular input line entry system) SMILES string representations of the drugs, and good results have been achieved. The PADME [13] model combines molecular graph convolution of compounds and protein features and uses fixed-rule descriptors to represent proteins, improving the predictive performance of the model. The model is more scalable than traditional machine learning models. WideDTA [17] builds on DeepDTA [8] by representing drugs and proteins as words, learning more potential characteristics of drugs and proteins. However, since the convolution window of a CNN is fixed, this network is unable to extract the features of contextual information. To represent molecules in a natural way that preserves as much molecular structure information as possible, thus allowing the model to better learn the relevance of the underlying space, an increasing number of approaches are utilizing graph neural networks to predict DTA. MT-DTI [18] introduces the attention mechanism in drug representation and takes more account of the correlation between different molecules, which improves the prediction performance of DTA and greatly increases the interpretability. In DeepGS [19], the topological structure information of a drug is extracted by using a graph attention network (GAT) [20], while the local chemical background of the drug is captured by using a bidirectional gated recurrent unit (Bi-GRU) [21] and combined with the protein sequence features extracted by a CNN for prediction. rzMLP [22] uses a gMLP model to aggregate input features with constant size, and uses a ReZero layer to smooth the training process for that block. The model is able to learn more complex global features while avoiding poor predictions due to a too deep model. EnsembleDLM [23] aggregates predictions from multiple deep neural networks, not only obtaining better predictions, but also exploring how much data deep learning networks need to achieve better prediction performance. GANsDTA [24] employs a generative adversarial network (GAN) [25] to extract features of protein sequences and compound SMILES in an unsupervised manner. Because GAN’s feature extractor does not require labeled data, the model is able to accommodate unlabeled data for training. Because GAN’s feature extractor does not require labeled data, the model is able to accommodate unlabeled data for training. The model can use more datasets to learn protein and drug features, thus achieving correspondingly better feature representation and prediction performance. GraphDTA [26] modelled drugs as molecular graphs with one-dimensional drug sequences, then put the graph into several graph network models and obtained deep learning models, which were excellent at the time. GraphDTA [26] demonstrated that representing drugs as graphs can further improve the prediction capabilities of deep learning models in terms of DTA.

**Table 1:** Prediction methods

Method	Published time	Model	Summary
SimBoost [11]	2016	Gradient boosting regression trees	Predicting continuous values of binding affinities of compounds and proteins
KronRLS [16]	2018	Multiple kernel learning	The first method for time- and memory-efficient learning with multiple pairwise kernels
DeepDTA [8]	2018	CNN	Processing protein sequences and compound 1D representations using convolutional neural networks
PADME [13]	2018	DNN	The first to combine Molecular Graph Convolution for compound featurization with protein descriptors
WideDTA [17]	2019	CNN	Combining four different textual pieces of information related to proteins and ligands
MT-DTI [18]	2019	Transformers + CNN	Proposing a new molecule representation based on the self-attention mechanism
GANsDTA [24]	2019	GAN + CNN	Effectively learning valuable features from labeled and unlabeled data
DeepGS [19]	2020	GAT + Bi-GRU	Extracting the topological information of the molecular map and the local chemical context of the drug
rzMLP [22]	2021	gMLP + ReZero	Use MHM block for multiple protein and ligand representations and rzMLP block to aggregate concatenated protein-ligand pair representations
EnsembelDLM [23]	2021	Multiple deep networks	Aggregating predictions from multiple deep neural networks
GraphDTA [26]	2021	GIN + CNN	Introducing multiple models of graph neural networks

However, two problems remain that prevent accurate DTA. (1) The GAT model used by some contemporary methods is a restricted form of static attention, and the attention coefficient function of the nodes in the drug graph is monotonic, which leads to the inability to comprehensively extract drug features. (2) When processing protein sequences, the contextual association information of amino acid sequences is not acquired, and the protein association features are thus ignored.

To solve the above problem, this paper proposes a method named dynamic graph DTA (DGDTA). In DGDTA, each drug is considered a graph of interactions between atoms and edges, and a dynamic attention score is used to consider which atoms and edges in the drug graph play more critical roles in predicting DTA. Compared with static attention, DGDTA is able to extract a more comprehensive drug signature. To better obtain the contextual features of amino acid sequences in proteins, DGDTA introduces bidirectional long short-term memory (Bi-LSTM) [27] to extract more comprehensive amino acid sequence features in combination with drugs. Through validations conducted on the Davis [28] and KIBA [14] datasets, DGDTA achieves better performance than the competing methods in terms of results. In this paper, a dynamic graph attention network example is given to further improve the representativeness and effectiveness of drug molecule maps. The experimental results demonstrate the effectiveness of DGDTA.

## Methods

**Fig. 1:**
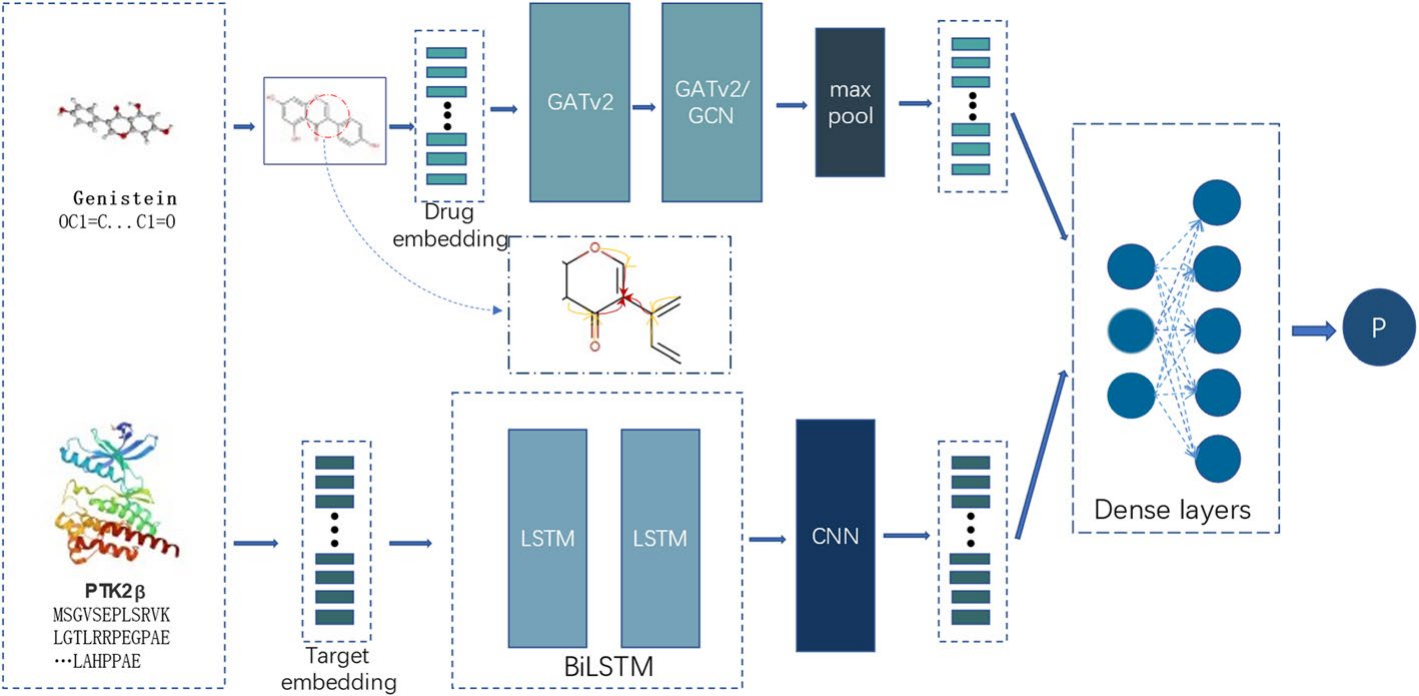
General architecture of DGDTA

DGDTA is a method for predicting DTA based on a deep learning network, and its architecture (shown in Fig. 1), is divided into three main steps. (1) Obtaining drug features. DGDTA uses the SMILES [29] as the drug compound input, and transforms the drug into a drug graph consisting of atoms and edges with reference to the natural properties of the drug. According to the literature, a two-layer graph network structure has better feature extraction performance. DGDTA uses a two-layer dynamic graph attention network (GATv2) [30] and a combination of GATv2 and a graph convolutional network (GCN) to obtain drug graph features, and DGDTA is divided into two versions: DGDTA-AL and DGDTA-CL. (2) Extracting protein features. DGDTA uses a combination of Bi-LSTM and multilayer convolutional networks to obtain more comprehensive protein amino acid sequence information while considering the contextual relationships among the amino acid sequences. (3) Performing DTA prediction. The observed connections among drug features and protein features during extraction used to determine DTA via a fully connected layer. The details of DGDTA are described in the following parts.

### Obtaining drug features

With the development of graph neural networks for DTA, many approaches have been presented. When using a graph to represent a drug, it is difficult to accurately extract graph features due to the complexity of drug graphs. DGDTA adopts a dynamic GAT to obtain drug features. Through SMILE code, drug’s atomic composition, and the valence charge number of atoms can be inferred, which can further judge drug information such as the number of hydrogen bonds, and then used for the drug’s feature representation in affinity prediction. To better extract drug features, DGDTA uses the SMILES [29] sequences of drugs as inputs, and uses RDKit to extract the atoms and interactions from the SMILES sequences. Then, DGDTA constructs a graph for each drug based on its SMILES sequence. A drug graph is denoted as $G=(V,E)$, where $V$ is a node represented by a drug atom, and $E$ represents the set of edges between nodes. Each node is represented by an n-dimensional vector from DeepChem [31]. This n-dimensional vector includes the atomic symbols, the number of adjacent hydrogen atoms, the number of adjacent atoms, the implicit valence of the atoms (implicit valence) and whether the bonds are aromatic. One node is represented as $\text{d}=\left\{{\text{f}}_{1},{\text{f}}_{2},{\text{f}}_{3}\dots ,{\text{f}}_{n}\right\}$. By representing the atoms d of each drug as the vertices of the drug graph, the features $D=\left\{{\text{d}}_{1},{\text{d}}_{2},{\text{d}}_{3}\dots ,{\text{d}}_{D}\right\}$ of each drug are obtained. To obtain more information about the graph structure in n-dimensional space, this paper adopts a dynamic attention mechanism for the graph:

1 $$e\left({d}_{i},{d}_{j}\right)={a}^{T}LeakyReLU\left(W\left[{d}_{i}\parallel {d}_{j}\right]\right) \quad j\in {\mathcal{N}}_{\mathcalligra{i}}$$

$e\left( {d_{i} ,d_{j} } \right)$ denotes the importance of the features of neighbour node $j$ to node $i$, where ${\mathcal{N}}_{\mathcalligra{i}}$ represents the neighbours of node $i$, $a\in {\mathbb{R}}^{{2d}^{{\prime }}}$, $W\in {\mathbb{R}}^{{2d}^{{\prime }}\times d}$ are learned, and II denotes vector concatenation. Utilizing the $softmax$ function to normalize all neighbours, we can obtain the following attention function:

2 $${a}_{ij}=softmax\left(e\left({d}_{i},{d}_{j}\right)\right)=\frac{exp\left(e\left({d}_{i},{d}_{j}\right)\right)}{{\sum }_{k\in {\mathcal{N}}_{\mathcalligra{i}}}exp\left(e\left({d}_{i},{d}_{k}\right)\right)}$$

Combining Eqs. (1) and (2), the coefficients of attention are expressed as:

3 $${a}_{ij}=\frac{{a}^{T}LeakyReLU\left(W\left[{d}_{i}\parallel {d}_{j}\right]\right)}{{\sum }_{k\in {\mathcal{N}}_{\mathcalligra{i}}}exp\left({a}^{T}LeakyReLU\left(W\left[{d}_{i}\parallel {d}_{j}\right]\right)\right)}$$

After integrating the feature information of the neighbouring nodes, we apply the nonlinear parameter $\sigma$, to obtain the output features of each node:

4 $${d}_{i}^{{\prime }}=\sigma \left({\sum }_{j\in {\mathcal{N}}_{\mathcalligra{i}}}{a}_{ij}W{d}_{j}\right)$$

Nodes are represented as the weighted averages of their neighbouring feature vectors. To further solidify the learning process of dynamic graph self-attention and improve the learning effect, the attention is extended to multiheaded attention.

5 $${d}_{i}^{{\prime }{\prime }}=\sigma \left(\frac{1}{H}\sum _{h=1}^{H}{\sum }_{j\in {\mathcal{N}}_{\mathcalligra{i}}}{{a}_{ij}}^{h}{W}^{h}{d}_{j}\right)$$

$H$ independent attention mechanisms connect the semantic feature vectors of the nodes through Eq. (5), and obtain an updated drug feature representation ${\text{D}}^{\left(1\right)}=\left\{{\text{d}}_{1}^{\left(1\right)},{\text{d}}_{2}^{\left(1\right)},{\text{d}}_{3}^{\left(1\right)}\dots ,{\text{d}}_{D}^{\left(1\right)}\right\}$. Based on a combination of research and experiments, a two-layer graph network structure is able to obtain more accurate prediction results. First, the graph network in the second layer uses a dynamic graph neural network and obtains the drug feature representations ${\text{D}}^{\left(2\right)}=\left\{{\text{d}}_{1}^{\left(2\right)},{\text{d}}_{2}^{\left(2\right)},{\text{d}}_{3}^{\left(2\right)}\dots ,{\text{d}}_{D}^{\left(2\right)}\right\}$; this version is named DGDTA-AL. After many experiments and comparisons, the graph network in the second layer is replaced with a GCN, whose propagation rules are as follows:

6 $${H}^{(l+1)}=\sigma \left({\stackrel{\sim}{D}}^{-\frac{1}{2}}\stackrel{\sim}{A}{\stackrel{\sim}{D}}^{-\frac{1}{2}}{H}^{\left(l\right)}{W}^{\left(l\right)}\right)$$

${H}^{\left(l\right)}$ denotes the nodal feature matrix of ${l}^{th}$, where $\stackrel{\sim}{A}=A+I$, $A$ is the adjacency matrix, $I$ is the unit matrix, $\stackrel{\sim}{D}=D+I$, $D$ is the degree matrix, and $W$ is a trainable weight. A drug feature representation ${\text{D}}^{{\left(2\right)}^{{\prime }}}=\left\{{\text{d}}_{1}^{{\left(2\right)}^{{\prime }}},{\text{d}}_{2}^{{\left(2\right)}^{{\prime }}},{\text{d}}_{3}^{{\left(2\right)}^{{\prime }}}\dots ,{\text{d}}_{D}^{{\left(2\right)}^{{\prime }}}\right\}$ is obtained. The GCN is applied to the full graph via the Laplacian matrix, which captures the connectivity relationships between the graph nodes and updates the node features of the full graph. In this paper, this version is named DGDTA-CL. We use the rectified linear unit ($ReLU$) activation function after each layer and use global maximum pooling in the last layer to obtain the vector representation of the drug.

### Extracting protein features

A protein sequence is a string of ASCII characters represented as amino acids. In many methods, one-hot codes are used to represent drugs and proteins, as well as other biological sequences, such as DNA and RNA. We use one-hot encoding to represent the atoms of the drug and incorporate atomic properties for drug initialization. Because drug molecules are shorter and simpler in structure than proteins, we utilize one-hot encoding to expand the dimensionality of the drug’s representation. This enables model to capture specific information associated with each drug atom. For protein, in order to prevent feature singularity, we employ different approaches for the initialization. In this paper, we map each amino acid to a numerical value and represent one protein as a sequence of integers. And then an embedding layer is added to the sequence, where each character is represented by a 128-dimensional vector. For training purposes, the sequences are cut or padded to a fixed sequence with a length of 1000. If the sequence is short, it is padded with 0 values. In this paper, the embedding representation ($\text{c}\in {\mathbb{R}}^{{\mathcalligra{d}}_{p}}$, where $d$ is the dimensionality of the protein embedding) is a Bi-LSTM layer that captures the dependencies the characters in a sequence of length $n$ ($C=\left[{\text{c}}_{1},{c}_{2}\dots {\text{c}}_{n}\right]$). We obtain ${p}_{i}\in {\mathbb{R}}^{{2\mathcalligra{d}}_{1}}$, where ${d}_{1}$ denotes the number of output cells used in each LSTM cell.

7 $$\overrightarrow{{p}_{i}}= \overrightarrow{LSTM}({\text{c}}_{\text{i}},{p}_{i-1})$$

8 $$\overleftarrow{{p}_{i}}=\overleftarrow{LSTM}({c}_{i},{p}_{i+1})$$

9 $${p}_{\text{i}}= \overrightarrow{{p}_{i}}\parallel \overleftarrow{{p}_{i}}$$

The vector $P$ is composed of the output vectors generated by the Bi-LSTM; i.e., $P=\left[{p}_{1},{p}_{2}\dots {p}_{n}\right]$. Finally, we use a one-dimensional convolutional layer to learn different levels of abstract features to obtain a vector of protein sequences representations.

### Performing DTA prediction

The prediction layer connects the learned drug vector representation with the vector representation of the protein sequence. Then, they are used as inputs and the output $y$ is obtained from the fully connected layer.

10 $$y={W}_{output}\left[D,P\right]+{b}_{output}$$

where ${W}_{output}$ denotes the weight matrix of the fully connected layer and ${b}_{output}$ denotes the bias of the fully connected layer.

We choose the mean square error (MSE) loss as the loss function, which has the advantage of a function curve that is smooth, continuous and derivable everywhere, making it convenient for use in the gradient descent algorithm. As the error decreases, the gradient also decreases, which is more conducive to convergence and more stable.

11 $$MSE=\frac{1}{n}\sum _{i=1}^{n}{({Y}_{i}-{y}_{i})}^{2}$$

where ${Y}_{i}\in {\mathbb{R}}^{\text{B}}$, ${y}_{i}\in {\mathbb{R}}^{\text{B}}$ denotes the predicted affinity value between the $i$th sample and the label of the affinity value in the sample, and $\text{B}$ denotes the batch size.

### Model training

DGDTA takes drug SMILES strings and protein amino acid sequences as inputs. In this paper, Python 3.9, PyTorch 1.12.1 and PyG2.1 are used to implement dynamic GAT and LSTM. In this paper, the number of layers in the graph neural network is set to 2, Bi-LSTM is applied, the number of hidden states is set to 10, and the dropout parameter is set to 0.2. Then, the proposed method is trained on the above dataset for 1000 epochs, and the adaptive moment estimation (Adam) optimizer is used with a learning rate of 0.0005. The devices that are used for the experiments are an Intel(R) Xeon(R) Platinum 8260 CPU @ 2.30 GHz and an NVIDIA GeForce RTX 3090 GPU.

## Results

In this section, we present the dataset used, the evaluation metrics, an ablation study and the results of a comparison with state-of-the-art methods. This section also illustrates the advantage of the dynamic GAT and gives an example of a real drug–target combination.

### Dataset and evaluation metrics

We use the Davis [28] and KIBA [14] datasets to evaluate the performance of the method proposed in this paper. The numbers of drugs and targets in the dataset, and the sample sizes for training and testing during the experiments are shown in Table 2. In this paper, the concordance index (CI; the larger the better) [32] and MSE (the smaller the better) are also used as the main indicators for evaluating the performance of the tested models. In this paper, the GAT and GAT_GCN models are chosen as baseline1 and baseline2 of the ablation study, respectively.

**Table 2:** Datasets

	Davis	KIBA
Drugs	72	2116
Targets	442	229
Total samples	30,056	118,254
Train samples	25,046	98,545
Test samples	5010	19,709

### Ablation study

**Fig. 2:**
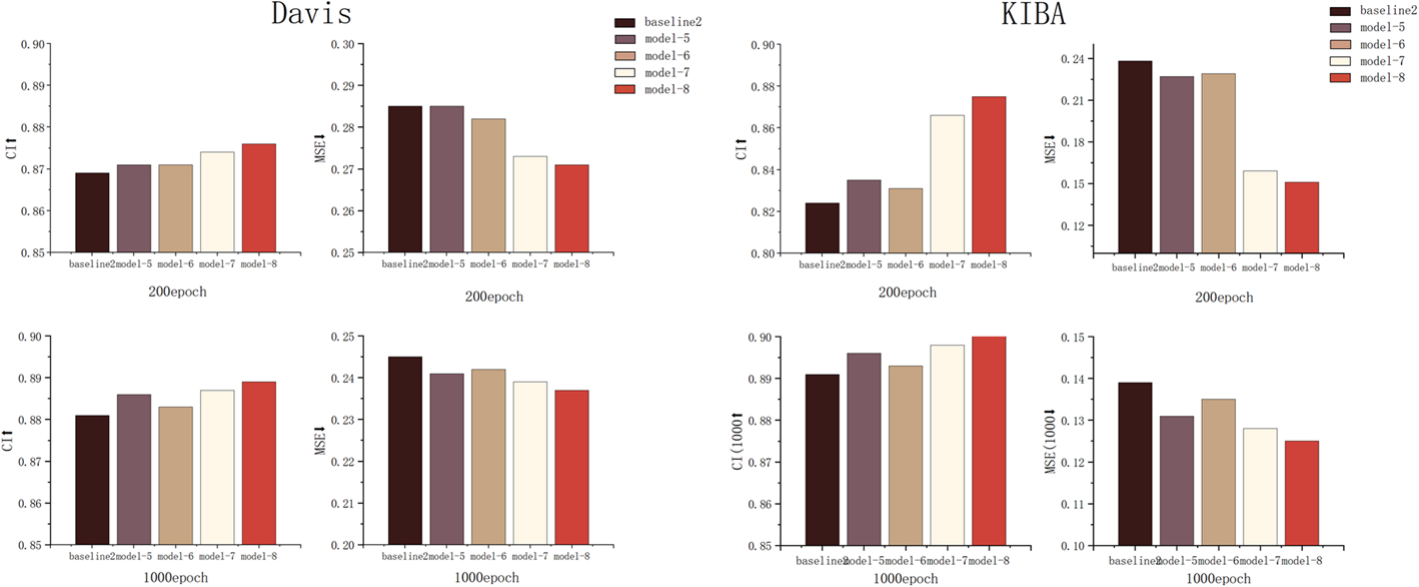
Comparison between baseline1 and different models at 200 and 1000 epochs

**Fig. 3:**
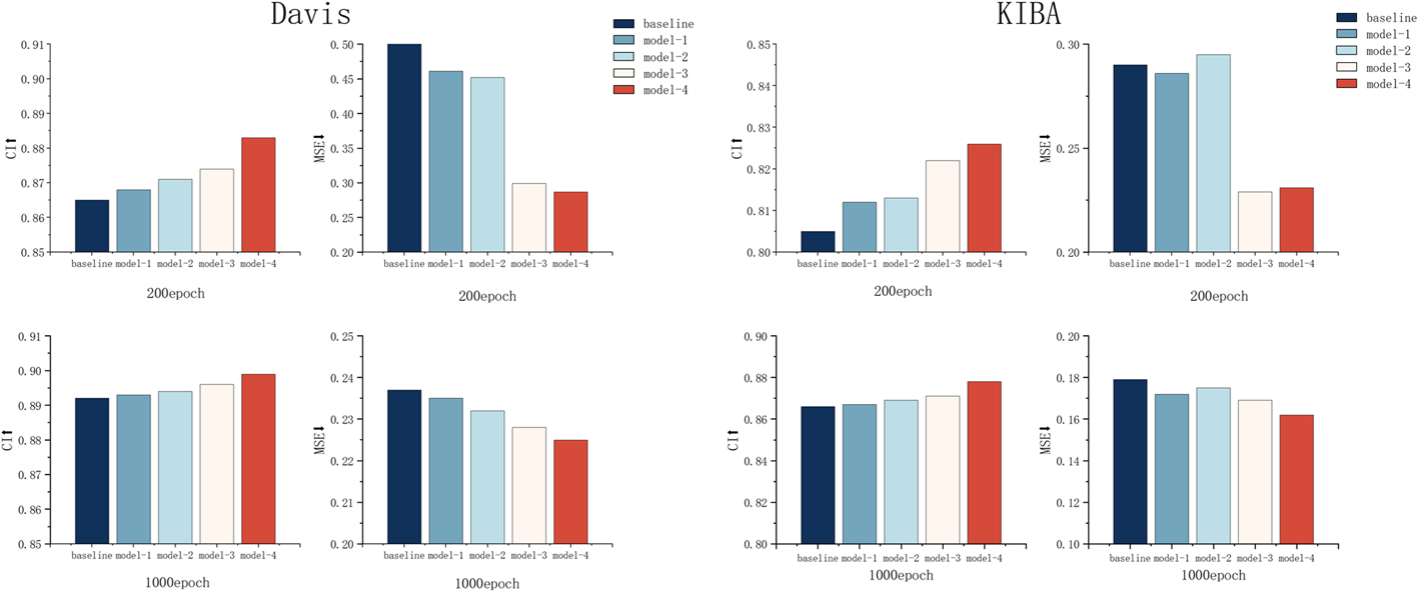
Comparison between baseline2 and different models at 200 and 1000 epochs

In the ablation study, we analyse the effectiveness of the innovative elements of our method. In this section, to be as fair as possible, we use the same training and testing sets as those employed by the baselines and the same evaluation metrics. In this paper, a dynamic graph neural network is incorporated into the drug graph, and Bi-LSTM is added to extract protein amino acid sequence features to further improve the model accuracy. The popular GRU model is added as a comparison method. GRU and LSTM are important variants of recurrent neural networks, and they have strong memory and long-distance dependence capturing ability when processing sequence data. GRU has higher computational efficiency with reduced parameter settings compared to LSTM, but this also leads to some loss of information at longer distances in some cases. In order to better capture the contextual association information of amino acid sequences and further prove the effectiveness of LSTM method, GRU is introduced as a comparison in the ablation study. And the results of the ablation study are shown in Figs. 2 and 3.

Figure 2 shows that on the Davis and KIBA datasets, the DTA prediction results obtained by Model-2 using the dynamic GAT achieve a higher CI and a smaller MSE than those of baseline 1 in the same number of epochs. Model-1 with the addition of Bi-LSTM method is also better than baseline1. Based on Model-2, Bi-LSTM is used to improve the ability to extract contextual protein amino acid sequence features. The evaluation score of Model-4 is improved further, while the prediction result is better than that of the GRU in Model-3 with the same parameters. Model-4 achieves the best results in the 200-epoch and 1000-epoch comparisons conducted on both datasets, and Model-4 is the DGDTA-AL method illustrated in 2.1. As shown in Fig. 3, Model-8 obtains the highest CI and the lowest MSE in the comparison with baseline 2 over the same number of epochs; Model-8 is the DGDTA-CL method.

In this paper, the results obtained by different models in the ablation study are presented in Table 3. On the Davis dataset, DGDTA-AL achieves the best results (in bold), reaching 0.899 and 0.225 CI and MSE values, respectively, which are improvements of 0.7% and 0.7% over those of baseline. DGDTA-CL achieves a CI of 0.902 and an MSE of 0.125 on the KIBA dataset, which are improvements of 1.1% and 1.4% over those of baseline 2, respectively. The results of the ablation study demonstrate the effectiveness of the innovative elements proposed in this paper.

**Table 3:** Ablation study on the Davis and KIBA datasets

Dataset	Methods	GATv2	GCN	GRU	LSTM	CI	MSE
Davis	Baseline1	–	–	–	–	0.892	0.232
	Model-1	–	–	–	✓	0.893	0.230
	Model-2	✓	–	–	–	0.895	0.232
	Model-3	✓	–	✓	–	0.896	0.228
	Model-4	✓	–	–	✓	0.899^*^	**0.225**
	Baseline2	–	✓	–	–	0.881	0.245
	Model-5	–	✓	–	✓	0.886	0.241
	Model-6	✓	✓	–	–	0.883	0.242
	Model-7	✓	✓	✓	–	0.887	0.239
	Model-8	✓	✓	–	✓	0.889	0.237
KIBA	Baseline1	–	–	–	–	0.866	0.179
	Model-1	–	–	–	✓	0.867	0.172
	Model-2	✓	–	–	–	0.869	0.175
	Model-3	✓	–	✓		0.871	0.169
	Model-4	✓	–	–	✓	0.878	0.162
	Baseline2	–	✓	–	–	0.891	0.139
	Model-5		✓	–	✓	0.896	0.131
	Model-6	✓	✓	–	–	0.893	0.135
	Model-7	✓	✓	✓	–	0.898	0.128
	Model-8	✓	✓	–	✓	**0.902**	**0.125**

*Bold values represent the best result

### Comparison with the state-of-the-art methods

**Fig. 4:**
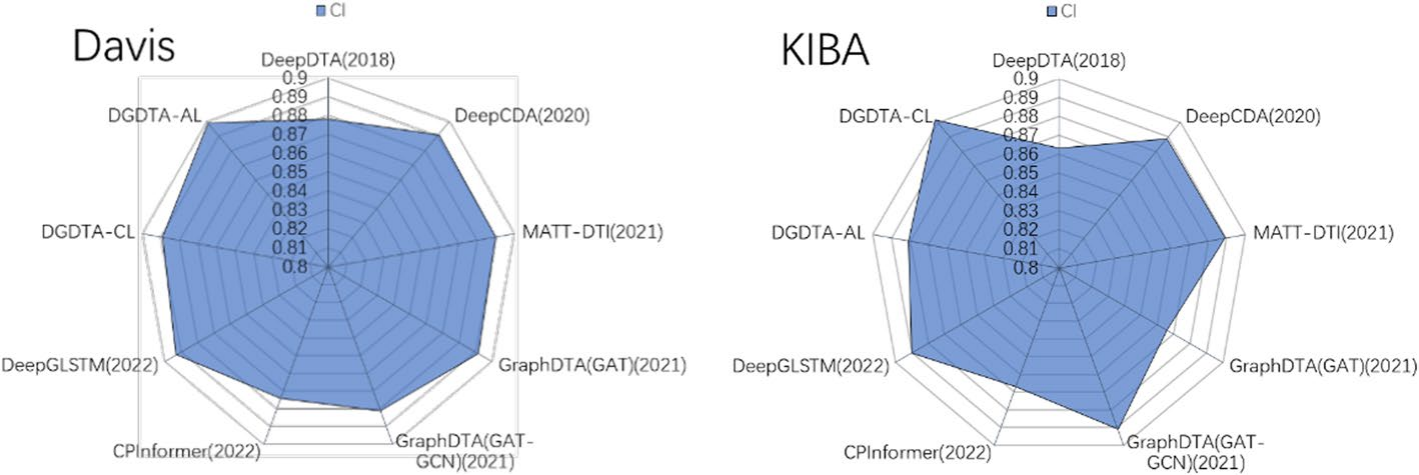
CI comparison among the experimental methods on the Davis and KIBA datasets

In this section, Table 4 shows the experimental results obtained by DGDTA and the comparison methods. To be consistent with the ablation experiment in 3.2, we use the same datasets and evaluation metrics. Based on this, we added the ${r}_{m}^{2}$ evaluation metric. As shown in Table 4, DGDTA-AL is better than the mainstream DTA methods in terms of the CI, MSE and ${r}_{m}^{2}$ on the Davis dataset. Compared with DeepGLSTM [33], which has the best results among the comparison methods, the CI and MSE of the proposed approach are improved by 0.6% and 1.1%, respectively. Additionally, the CI and MSE are improved by 0.9% and 0.4%, respectively, over those of the excellent MATT-DTI [34] method. And, ${r}_{m}^{2}$ reaches 0.707. As shown in Table 4, DGDTA-CL achieves a more significant improvement in its results on the KIBA dataset. Compared with the DeepGLSTM [33] method, DGDTA-CL attains 1.2% and 1.8% performance improvements in terms of the CI and MSE metrics, and 1.3% and 2.5% CI and MSE improvements are achieved over the MATT-DTI [34] method, respectively. And, ${r}_{m}^{2}$ reaches 0.809. Figure 4 plots the CI scores obtained by the methods in the table for both datasets to further demonstrate the performance improvement provided by the DGDTA method. The experimental results show that DGDTA is better than the comparative methods, and the use of a dynamic graph with attention to extract drug features and effective contextual protein information is significant for predicting DTA.

**Table 4:** Comparison with the state-of-the-art methods

Dataset	Methods	CI	MSE	${\textbf{r}}_{\textbf{m}}^{2}$
Davis	DeepDTA [8]	0.878	0.261	0.631
	DeepCDA [35]	0.891	0.248	0.652
	MATT-DTI [34]	0.890	0.229	0.688
	GraphDTA(GAT) [26]	0.892	0.232	0.689
	GraphDTA(GAT-GCN) [26]	0.881	0.245	0.667
	CPInformer [6]	0.874	0.277	0.621
	DeepGLSTM [33]	0.893	0.236	0.679
	DGDTA-CL (ours)	0.889	0.237	0.672
	DGDTA-AL (ours)	0.899^*^	**0.225**	**0.707**
KIBA	DeepDTA [8]	0.863	0.194	0.673
	DeepCDA [35]	0.889	0.176	0.682
	MATT-DTI [34]	0.889	0.150	0.762
	GraphDTA(GAT) [26]	0.866	0.179	0.738
	GraphDTA(GAT-GCN) [26]	0.891	0.139	0.789
	CPInformer [6]	0.867	0.183	0.678
	DeepGLSTM [33]	0.890	0.143	0.789
	DGDTA-AL (ours)	0.881	0.162	0.762
	DGDTA-CL (ours)	**0.902**	**0.125**	**0.809**

*Bold values represent the best result

### Advantages of the DGDTA model

**Fig. 5:**
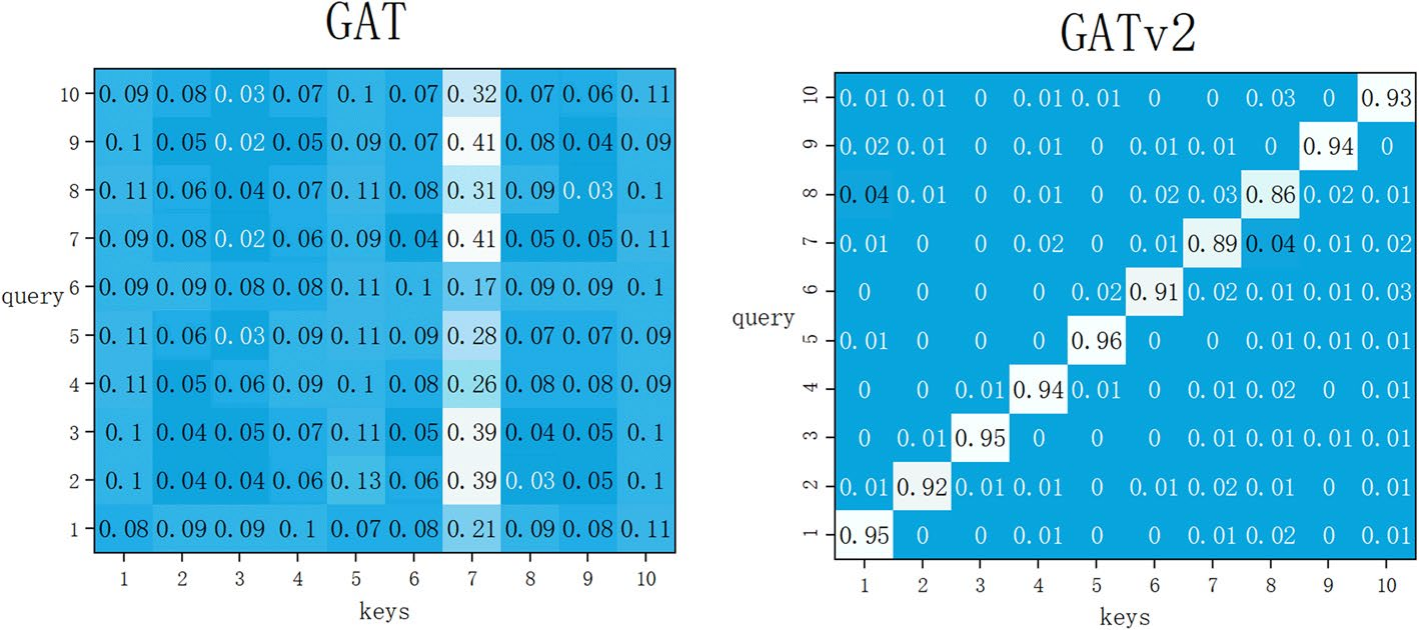
Attention coefficients of the GAT and GATv2

A dynamic GAT suggests that a traditional GAT is only a computationally constrained form of “static” attention: for any query node, the attention function is monotonic with respect to the key fraction [30]. As shown in the GAT heatmap presented in Fig. 5, the ordering of the attention coefficients is global, and all queries focus primarily on the 7th key.

12 $$e\left({d}_{i},{d}_{j}\right)=LeakyReLU\left({a}^{T}\left[W{d}_{i}\right]\parallel \left[W{d}_{j}\right]\right) j\in {\mathcal{N}}_{\mathcalligra{i}}$$

Formula (10) is the method for calculating the attention coefficients in the GAT, indicating the importance of the feature of node $j$ to node $i$. As ${\mathcal{N}}_{\mathcalligra{i}}$ is limited, there exists $a$ node ${j}_{max}$ where the attention distribution $a$ only calculates static attention from ${j}_{max}$ due to it being the maximum value. To overcome the monotonicity restriction of the key score, Formula (12) is transformed into Formula (1). This variant is more expressive than the GAT, as shown in the attention maps of GATv2 in Fig. 5. Since static attention cannot have different correlations for different keys and different queries, if there is one key that has a higher attention score than the others, then no query can ignore the score of this key, which results in very limited static attention.

**Fig. 6:**
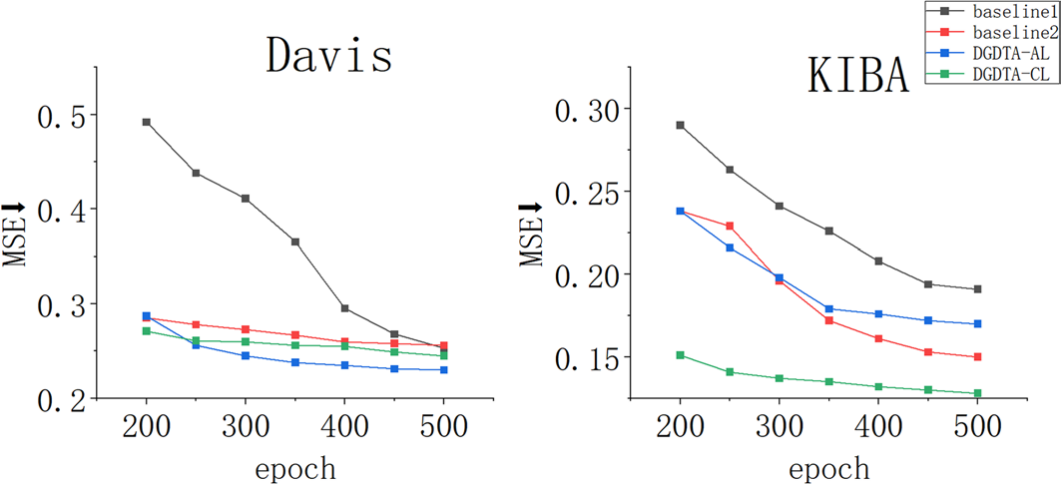
MSE trend

Among the datasets, Davis contains 2457 positive samples and 27,599 negative samples, the total number of samples is small, and the label distribution in the dataset is unbalanced. KIBA has 22,729 positive samples and 95,525 negative samples, so it contains more samples than Davis, but most of the labels in KIBA are very concentrated, and the label distribution is relatively normal. These problems create barriers for the model in terms of affinity prediction. Dynamic graph attention pays different amounts of attention to different queries in the attention score, enabling it to better distinguish the similarities and differences between samples. It is more discriminative during drug graph extraction and alleviates the imbalance problem in the given dataset. Figure 6 shows the MSE changes exhibited by the DGDTA-AL, DGDTA-CL, baseline 1 and baseline 2 models on Davis and KIBA at 200 and 500 epochs. Blue and green represent our proposed models with faster decreasing trends. The results demonstrate the more significant improvement yielded by the dynamic GAT in terms of predicting DTA.

### Example of a realistic drug–target combination

**Fig. 7:**
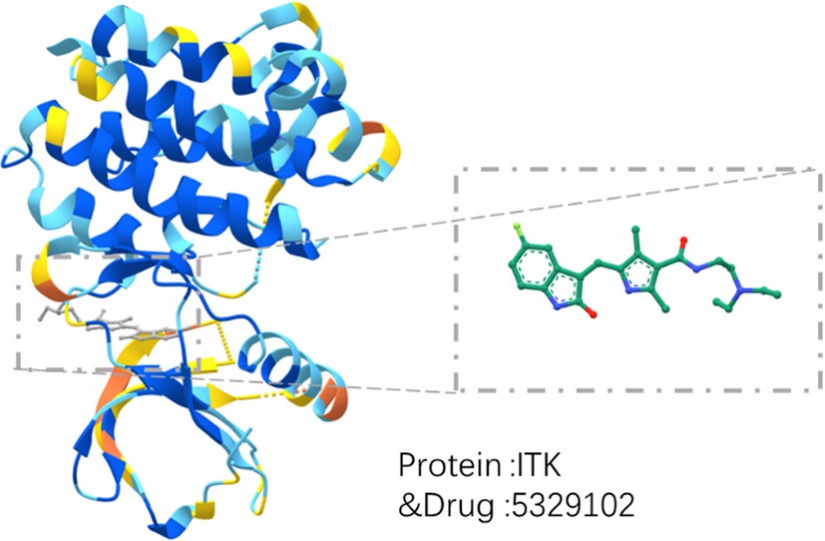
Visualization of the binding of a drug ‘DB5329102’ and a target ‘ITK’

To further demonstrate the validity of the proposed method, this paper gives an example to show the 3D model produced for a tested sample in reality. As shown in Fig. 7, the targeted drug (sunitinib) inhibits receptor tyrosine kinases (RTKs), where certain receptor tyrosine kinases are involved in tumour growth, pathological blood vessel formation and tumour metastasis. In biological and cytometric assays, sunitinib has been shown to inhibit tumour growth, cause tumour regression and inhibit tumour metastasis. In this paper, the bound small drug molecules are scaled up on the right side, and the drug and its binding target correspond to the drug ‘DB5329102’ and the target ‘ITK’ in the test set, respectively; this is done to verify the validity and practicality of the model proposed in this paper in practical applications through known drug–target binding examples.

## Discussion

In this paper, DGDTA is proposed based on the dynamic graph attention model and is divided into two versions, DGDTA-AL and DGDTA-CL, to predict the affinity values between drugs and proteins. Ablation experiments are performed on the Davis and KIBA datasets, and the proposed approach is compared with the DTA models that are popular today. The experimental results show that DGDTA can achieve better prediction performance and demonstrate that the dynamic graph attention model can extract more comprehensive feature representations from molecular drug maps.

## Conclusions

DGDTA can effectively predict DTA via deep learning, and it can obtain high CI and MSE metrics on experimental datasets, but it still has shortcomings. First, while dynamic graph attention models attain good prediction performance, they also require increased prediction time and computational cost. Second, drugs and proteins have very complex spatial structures, and much characteristic drug and protein information is lost in one-dimensional sequences.

In the future, further consideration will be given to fusing other characteristic drug information, such as their side effects, physicochemical properties, and deep structures. This will contribute to improving the performance of drug–target binding prediction models from various aspects.

## Data Availability

The Davis and KIBA data can be downloaded from https://github.com/thinng/GraphDTA/tree/master. The software and sample result as part of this project are readily avail- able from GitHub at https://github.com/luojunwei/DGDTA. Project name: DGDTA. Project home page: https://github.com/luojunwei/DGDTA. Operating system(s): Linux or other unix-like systems. Programming language: python 3.x. License: GNU GPL v3. Any restrictions to use by non-academics: license needed.

## Abbreviations

DGDTA: Dynamic graph DTA
DTA: Drug–target binding affinity
GAT: Graph attention network
Bi-LSTM: Bidirectional long short-term memory
SMILES: Simplified molecular input line entry system
CNN: Convolutional neural network
Bi-GRU: Bidirectional gated recurrent unit
GAN: Generative adversarial network
GATv2: Dynamic graph attention network
RTK: Receptor tyrosine kinases
GCN: Graph convolutional network
